# Identification, Comparison and Classification of Volatile Compounds in Peels of 40 Apple Cultivars by HS–SPME with GC–MS

**DOI:** 10.3390/foods10051051

**Published:** 2021-05-11

**Authors:** Shunbo Yang, Nini Hao, Zhipeng Meng, Yingjuan Li, Zhengyang Zhao

**Affiliations:** 1 College of Horticulture, Northwest A & F University, Yangling 712100, China; yangshunboa@163.com (S.Y.); haonini66@163.com (N.H.); 15035600947@163.com (Z.M.); l15536281655@163.com (Y.L.); 2 Apple Engineering and Technology Research Center of Shaanxi Province, Yangling 712100, China

**Keywords:** volatile compounds, peels, apple cultivars, HS-SPME, GC-MS, PCA

## Abstract

Aroma is an important quality indicator for apples and has a great influence on the overall flavour and consumer acceptance. However, the information of the aroma volatile compounds in apple peels is largely unknown. In this study, evaluation of volatile compounds in peels of 40 apple cultivars was carried out using headspace solid-phase microextraction (HS-SPME) coupled with gas chromatography-mass spectrometry (GC-MS). A total of 78 volatile compounds were identified, including 47 esters, 12 aldehydes, 5 alcohols, 3 ketones, 1 acid and 10 others. Eight volatile compounds were common in all apple cultivars. Cultivar Changfu No. 2 contained the highest number of volatile compounds (47), while Qinyue contained the least (20). Honey Crisps had the highest volatile content, at 27,813.56 ± 2310.07 μg/kg FW, while Huashuo had the lowest volatile content, at 2041.27 ± 120.36 μg/kg FW. Principal component analysis (PCA) clustered the 40 apple cultivars into five groups. Aroma is cultivar-specific, volatile compounds of hexyl butyrate, hexyl 2-methylbutyrate and hexyl hexanoate, together with hexanal, (E)-2-hexenal, 1-hexanol, estragole and α-farnesene could be proposed for apple cultivar classification in the future.

## 1. Introduction

Aroma, which is one of the most important quality indicators for fruits, has a great influence on the overall flavour and consumer acceptance [1]. It is generally a complex mixture of volatile compounds whose composition and concentrations are specific to the species, and often the variety, of fruit [2,3]. Volatile compounds, which determine the aroma profile of fruits, directly contribute to perceived odour and flavour attributes. Knowledge of these volatile compounds forms the basis of breeding programs aiming to improving fruit aroma. As an important trait of fruit quality, more attention has been paid to the study of aroma volatiles in recent years.

Apples (*Malus*×*domestica* Borkh.) are one of the most widely cultivated and frequently consumed fruits in the world [4]. Aroma is an important standard for evaluating the quality and characteristics of apples, and the aroma volatile compounds in apples have been studied for more than 50 years. Although more than 300 volatile compounds have been identified in apples, including alcohols, aldehydes, acids, ketones, terpenoids, sesquiterpenes, and esters, only a subset of 20–30 compounds significantly contribute to the typical apple aroma [5,6]. Among these, esters are the most abundant compounds. The esters, especially those with even-numbered carbon chains including combinations of acetic, butanoic, and hexanoic acids with ethyl, butyl, and hexyl alcohols, are the major contributors to apple volatiles. Butyl acetate, hexyl acetate, 2-methylbutyl acetate, and ethyl 2-methyl-butanoate are the crucial esters due to their high content and impact on the aroma of several apple varieties [7]. Alcohols are another important group of compounds, after esters, which affect the aroma of ripe apples, with the most abundant being 2-methyl-1-butanol, 1-butanol, 1-hexanol and 1-propanol [8,9]. Aldehydes are abundant in pre-climacteric apples, but after ripening, some aldehydes become almost imperceptible [10]. More than 25 aldehydes, mostly hexanal, trans-2-hexenal, and butanal, have been identified in apples [8]. During apple ripening, the volatile compounds are converted from aldehydes to esters to such an extent that esters can account for more than 80% of all aromatic compounds in some cultivars, such as Golden Delicious and Golden Reinders [9,11]. Aroma is cultivar-specific; therefore, study of the volatile profile at the variety level is necessary. Volatile compounds have been investigated at the germplasm level for peach (*Prunus persica*), pear (*Pyrus ussuriensis*), and melon (*Cucumis melo*) [12,13,14]. However, there are few studies on the comparative analysis of volatile compounds in a number of apple cultivars.

There are some microextraction techniques for the determination of volatile compounds, such as continuous sample drop flow microextraction [15], dispersive liquid–liquid microextraction [16] and solid-phase microextraction (SPME) [17]. The determination of volatile compounds in apples requires a suitable selective, sensitive analytical method. Although the lifetime of the microfiber is short, SPME, a simple, solvent-free method for the extraction of volatile compounds, combined with gas chromatography-mass spectrometry (GC-MS), has been widely used for the qualitative and quantitative analysis of volatile compounds in apple fruit [18,19].

In this study, HS-SPME combined with GC-MS was used to determine the composition and concentration of the volatile compounds in 40 apple cultivars. This work evaluated the aroma profiles of apple peels at cultivar levels, and these results could be valuable for future breeding programs, aiming to produce apple cultivars with enhanced aroma quality.

## 2. Materials and Methods

### 2.1. Plant Materials

**Figure 1:**
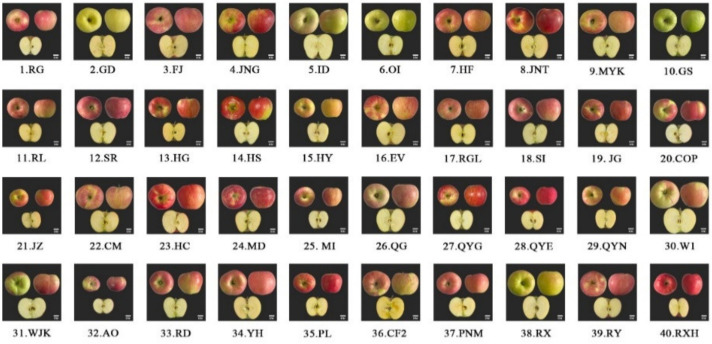
Materials of 40 apple cultivars used in this study. The codes refer to third column of Table 1. Bars = 2 cm.

The 40 apple cultivars used in this study are listed in Figure 1 and Table 1, along with some basic compositional parameters. The apples were harvested in 2019 from the experimental station of Northwest A and F University, Baishui County, Shaanxi Province, China (35°21′ N, 109°55′ E). Orchard management procedures such as irrigation, pruning, disease control and fertilisation, were similar for all cultivars. Fruits were sampled at full ripening and maturity was determined by taste, ground colour, starch index and days after pollination. Three biological replicates from three trees of each cultivar were prepared, with 4–6 fruits per replicate. Fruit peels (<1 mm in thickness) were collected from each apple with an apple peeler, immediately frozen in liquid nitrogen, and stored at −80 °C until analysis.

### 2.2. Physiological Characteristics Measurement

Single fruit weight was measured by an electronic balance (Mettler-Toledo Inc., Greifensee, Switzerland). The apple fruits’ total soluble solid (TSS) and titratable acidity (TA) was determined by a hand refractometer (Atago, Tokyo, Japan) and a digital fruit acidity meter (GMK-835F Perfect, Berlin, Germany), respectively.

### 2.3. HS-SPME Procedure

HS-SPME was applied for the extraction and concentration of volatile compounds in apple peels. All the extractions were performed using a divinylbenzene/carboxen/polydimethylsiloxane (DVB/CAR/PDMS) fibre with a thickness of 50/30 μm (Supelco, Bellefonte, PA, USA). For the extraction of volatile compounds, 5 g of apple peel was placed into a 50 mL screw-cap headspace vial containing a magnetic stirring rotor and 1 g NaCl spiked with 10 μL (0.4 mg/mL) 3-nonanone (internal standard). Subsequently, the headspace bottle was equilibrated at 50 °C for 10 min on a metal heating platform with agitation. Prior to use, the new SPME fibre was conditioned in the GC injector port for 0.5 h at 240 °C. Then, the fibre was inserted into the headspace with continuous heating and agitation (200 rpm) for 30 min to adsorb volatile substances. After extraction, it was introduced into the heated injector port of the chromatograph for desorption at 250 °C for 2.5 min.

### 2.4. GC-MS Analysis

A Thermo Trace GC Ultra gas chromatograph (Agilent Technologies Inc., Palo Alto, CA, USA) equipped with an HP-INNOWax capillary column (60 m × 0.25 mm × 0.25 μm) was used for analysis. The oven temperature was programmed as follows: 40 °C held for 3 min, raised to 150 °C at 5 °C/min, then increased at 10 °C/min to 220 °C and held for 5 min. Helium, the carrier gas, was circulated at 1.0 mL/min at a constant flow rate in splitless mode. The temperature of the ion source and transfer line were both maintained at 240 °C. MS fragmentation was performed under an electron ionisation of 70 eV with the scan range of 35–450 *m/z*.

### 2.5. Qualitative and Semi-Quantitative Analysis

Xcalibur 3.2 software was used to process the data collected from the GC-MS. Volatile compounds were identified by comparing retention indices (RI) and retention times (RT) to those of compounds in the NIST/EPA/NIH Mass Spectral Library database (NIST, 2014). Linear retention indices were calculated under the same chromatographic conditions after injection of a C7-C30 n-alkane series (Supelco, Bellefonte, PA, USA). Based on the total ion chromatogram, the content of each volatile compound was quantified as 3-nonanone equivalent (internal standard) by the peak area.

### 2.6. Statistical Analysis

All the data were the mean of three replicates. Excel 2010 software was conducted for statistical analysis and charting of data. Principal component analysis (PCA) was executed using Origin 2017 software (OriginLab Corporation, Northampton, MA, USA).

## 3. Results and Discussion

### 3.1. Identification and Determination of Volatile Compounds in Forty Apple Cultivars

The identification of volatile compounds and studies of diversity among cultivars were performed based on the retention indices obtained from GC-MS. A total of 78 volatile compounds were identified and quantified in 40 apple cultivars, including 47 esters, 12 aldehydes, 5 alcohols, 3 ketones, 1 acid and 10 other compounds (Table 2). On average, 35 types of volatile compound were detected in each cultivar. Changfu No. 2 (CF2) contained the highest number of volatile compounds (47), while the Qinyue (QYE) contained contained the least (20) (Table 3). More than 40 types of volatile compound were identified in Ralls (RL, 45 types), Huayu (HY, 44 types), Modi (MI, 44 types) and Ruixianghong (RXH, 43 types). Fewer than 25 types of volatile compound were identified in Huashuo (HS, 21) and Cox Orange (COP, 24) (Table 3). Eight volatile compounds (E17 hexyl acetate, E26 butyl caproate, E27 hexyl butyrate, E28 hexyl 2-methylbutyrate, E40 hexyl hexanoate, A1 hexanal, A4 2-hexenal and O8 α-farnesene) were present in peels of all apple cultivars (Tables S1 and S2). As shown in Table 2, hexyl butyrate (E27), hexyl 2-methylbutyrate (E28), hexyl hexanoate (E40), and 2-hexenal (A4) and α-farnesene (O8) were the most abundant compounds (average content > 700 µg/kg FW) in the apple cultivars, which is in agreement with the results of previous studies [20,21,22].

Aroma is a complex mixture of many volatile compounds, and the amount and content of aroma substances showed different patterns among various apple cultivars [18,23]. In this study, differences were also observed in the total content of volatile compounds among the 40 apple cultivars, ranging from 2041.27 ± 120.36 μg/kg FW to 27,813.56 ± 2310.07 μg/kg FW (Table 3). Honey Crisps (HC) had the highest content of volatile compounds, followed by Jazz (JZ, 27,493.25 ± 3800.46 μg/kg FW) and RXH (27,015.38 ± 2540.92 μg/kg FW). In contrast, HS had the lowest volatile compound content, followed by COP (2622.09 ± 150.74 μg/kg FW) and Royal Gala (RG, 2919.26 ± 351.23 μg/kg FW). The total content of volatile compounds in Orin (OI, 16,863.94 ± 1806.24 μg/kg FW), Red General (RGL, 15,447.86 ± 1120.35 μg/kg FW), and Envy (EV, 13,286.84 ± 1139.54 μg/kg FW) were 3- to 4-fold greater than those in Granny Smith (GS, 3930.31 ± 328.94 μg/kg FW), Starkrimson (SR, 3784.77 ± 327.05 μg/kg FW), and Fuji (FJ, 3562.94 ± 310.02 μg/kg FW). The above analysis indicates that the volatiles were dependent, to a great extent, on the cultivars, which is consistent with a previous study [18]. Golden Delicious (GD) has been reported to have the high volatile compound content [24]. However, the total content of volatiles in cultivar GD in this study (4436.74 ± 425.36 μg/kg FW) was not high. This result might be attributed to geographical variations, such as territory, climate, water and other environmental factors.

### 3.2. Composition and Concentration of Volatile Compounds

**Figure 2:**
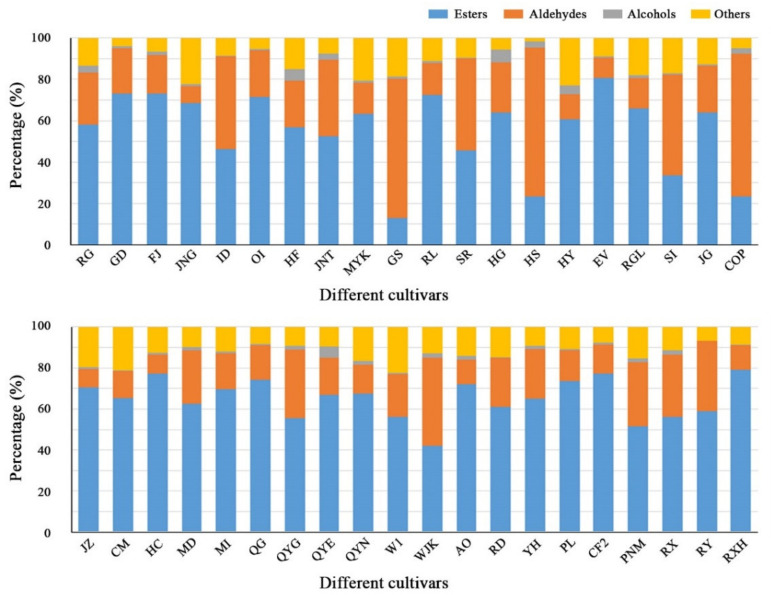
Percentage (%) of each type of volatiles in peels of 40 apple cultivars.

Esters, aldehydes, alcohols, ketones, acids and other volatiles constitute the aroma of different apple cultivars [2,5,18]. The composition and concentrations of volatile compounds in the peels of 40 apple cultivars are shown in Table S3. The percentage of each type of volatile in peels of 40 apple cultivars are presented in Figure 2 and Table S4. The total content of each type of volatile in apple cultivars are presented in Table 4.

#### 3.2.1. Esters

Esters are the dominant aromatic compounds in apples that form and contribute to the characteristic fresh and fruity apple flavour [29,30]. In this study, esters constituted the largest proportion of volatile compounds and 47 types were identified by SPME/GC-MS in the peels of 40 apple cultivars. HC had the highest ester content (21,457.95 ± 2230.10 μg/kg FW, 77.15% of total volatiles), followed by RXH (21,403.00 ± 2350.36 μg/kg FW, 79.23% of total volatiles), Qinguan (QG, 19,396.06 ± 2010.57 μg/kg FW, 70.39% of total volatiles) and JZ (19,352.58 ± 1523.65 μg/kg FW, 70.39% of total volatiles) (Table 4 and Table S4). By comparison, HS (474.23 ± 35.21 μg/kg FW), GS (504.68 ± 38.94 μg/kg FW) and COP (608.05 ± 52.84 μg/kg FW) had a lower ester content, accounting for 12.84%–23.23% of the total volatiles (Table 4 and Table S4). These results confirmed a previous observation that the volatile compound profile is highly cultivar-dependent, owing to the variation in esters, which is under strong genetic control [24].

In this study, the major ester compounds (average content > 100 μg/kg FW) were butyl acetate (E7), 2-methylbutyl acetate (E8), butyl butyrate (E14), butyl 2-methylbutyrate (E15), hexyl acetate (E17), hexyl propanoate (E23), butyl caproate (E26), hexyl butyrate (E27), hexyl 2-methylbutyrate (E28), hexyl hexanoate (E40) and butyl caprylate (E41) (Table 2), which was in agreement with the previous research [31,32]. Moreover, the most abundant esters (average content > 700 μg/kg FW) as determined by GC-MS were hexyl butyrate (E27), hexyl 2-methylbutyrate (E28) and hexyl hexanoate (E40). By comparison, ethyl propanoate (E2), propyl propionate (E5), propyl butyrate (E9), heptyl acetate (E25), ethyl octanoate (E29), 3-methylbut-2-enyl hexanoate (E39) and butyrolactone (E43) were present in relatively low amounts (average content < 3 μg/kg FW) in peels of each apple cultivar (Table 2).

Hexyl acetate, hexyl hexanoate, and hexyl 2-methylbutyrate, which are the most important esters, have a great influence on apple aroma because of their abundance [7,8]. Consistent with previous reports [33,34], hexyl 2-methylbutyrate (E28) was the most abundant in most of the cultivars analysed in this study, such as HC (10,087.55 ± 1534.58 μg/kg FW), JZ (8980.62 ± 850.45 μg/kg FW) and OI (7589.21 ± 865.32 μg/kg FW) (Table S3). Hexyl acetate has a sweet and fruity odour, with floral notes [35]. The contents of hexyl acetate (E17) in RXH (1399.58 ± 145.63 μg/kg FW) and EV (1380.88 ± 15.86 μg/kg FW) were higher than in the other apple cultivars. Conversely, the cultivars with the lowest content of hexyl acetate (E17) were Qinyun (QYN, 10.61 ± 1.85 μg/kg FW) and Ruixue (RX, 11.88 ± 1.02 μg/kg FW) (Table S3). Hexyl hexanoate is another main ester in apples [30]. HC, QG, and RXH had higher levels of hexyl hexanoate (E40), at concentrations of 5688.01 ± 415.97 μg/kg FW, 5494.48 ± 475.20 μg/kg FW, and 5403.15 ± 586.30 μg/kg FW, respectively, whereas Yuhuazaofu (YH), COP, and GS had lower levels, at 83.05 ± 7.64 μg/kg FW, 88.31 ± 9.20 μg/kg FW and 139.78 ± 12.96 μg/kg FW, respectively (Table S3). Moreover, hexyl butyrate (E27) was responsible for fruit and sweet aroma impressions and was detected in all apple samples, reaching 2709.52 ± 302.51 μg/kg FW in cultivar QG (Tables S1 and S3).

#### 3.2.2. Aldehydes

Aldehydes were the second most abundant volatiles in this study, accounting for between 8.25% (Jonagold, JNG) and 69.23% (COP) of the total volatile content in the apple cultivars (Figure 2; Table S4). More than 25 aldehydes have been identified in apples [8,36]. In this study, 12 types of aldehyde compound were identified (Table 2). Aldehyde content varied greatly among the apple cultivars and ranged from 662.21 ± 80.12 μg/kg FW (18.59% of total volatiles) in FJ to 4905.73 ± 520.41 μg/kg FW (22.60% of total volatiles) in Jiguan (JG) (Table 4; Table S4). Hexanal (A1) and (E)-2-hexenal (A4) were the most predominant constituent aldehydes (average content > 200 μg/kg FW) in all apple cultivars in this study, which was in agreement with a previous report [28].

Hexanal is an important contributor to the characteristic fish-like sweet odours and confers a green aroma to apples [22]. HY had the highest content of hexanal (A1), at 1050.38 ± 85.45 μg/kg FW, followed by JNG (756.56 ± 82.36 μg/kg FW) and QG (517.39 ± 45.28 μg/kg FW). In contrast, the hexanal content in Ruiyang (RY, 29.29 ± 1.86 μg/kg FW) and FJ (42.77 ± 8.65 µg/kg FW) was much lower than in other cultivars (Table S3). (E)-2-Hexenal confers a green leafy sensorial attribute to apple flavour [37]. The highest content of (E)-2-hexenal (A4) was 4435.22 ± 500.52 μg/kg FW in JG, while the lowest was 569.95 ± 26.95 μg/kg FW in FJ. In addition, nonanal was detected in 24 apple cultivars, providing a strong smell of grease and a sweet orange flavour [38]. On the other hand, (E,E)-2,4-Heptadienal (A9) was found only in cultivar Indo (ID) and GS (Table S3).

#### 3.2.3. Alcohols

Alcohols are another main group of compounds contributing to apple aroma in the 40 apple cultivars. Among the alcohols, 2-hexyn-1-ol (B4) and 1-hexanol (B5) were the major components. Five types of alcohol were identified. The relative cumulative content of alcohols ranged from 0.15% in cultivar RY to 6.00% in cultivar Huaguan (HG) (Table S4). HY (941.15 ± 80.34 μg/kg FW, 3.95% total volatiles) had the highest alcohol content, followed by HG (730.52 ± 50.46 μg/kg FW, 6.00% total volatiles) and Hanfu (HF, 581.55 ± 42.89 μg/kg FW, 5.29% total volatiles) (Table 4 and Table S4). In contrast, RY (6.27 ± 1.02 μg/kg FW, 0.15% total volatiles) has the lowest alcohol content, followed by SR (18.63 ± 2.63 μg/kg FW, 0.49% total volatiles) and Starking (SI, 26.48 ± 5.21 μg/kg FW, 0.49% total volatiles) (Table 4 and Table S4).

1-hexanol and 1-butanol are the most dominant alcohols identified in apples [39,40]. 1-Hexanol can suppress the apple-like odour due to an unpleasant and earthy odour, which contributes negatively to the apple aroma [41]. In this study, the highest content of 1-hexanol (B5) was observed in HG (393.09 ± 21.25 μg/kg FW), followed by HF (364.88 ± 42.56 μg/kg FW) and Alps Otome (AO, 343.74 ± 40.62 μg/kg FW) (Table S3). In contrast, 1-butanol is considered a positive contributor to the features of apple aroma due to its characteristic sweet aroma [39]. In this study, HY had a higher content of 1-butanol (B2) than the other apple cultivars, up to 542.07 ± 49.62 μg/kg FW (Table S3). In addition, 2-hexyn-1-ol (B4) was detected in 38 of the 40 apple cultivars (Table S1), with the highest content in QG (66.21 ± 5.21 μg/kg FW), followed by JG (58.55 ± 5.01 μg/kg FW) and GS (34.84 ± 2.69 μg/kg FW) (Table S3).

#### 3.2.4. Ketones, Acids and Other Compounds

There are 3 ketones, 1 acid and other 10 types of volatile, constituting 1.61%–23.12% of the total volatile substances (Table S4). Ketones have a floral and fruity sweet flavour [42]. The three ketone compounds detected in this study were 1-penten-3-one (C1), 1-octen-3-one (C2), and 6-methyl-5-hepten-2-one (C3). 2-Methylbutanoic acid (D1) was the only acid compound detected, with cultivar HY having the highest acid content (201.97 ± 12.55 μg/kg FW) (Table S3). Additionally, α-farnesene (O8) was detected in all apple peels, and ranged from 7.76 ± 0.85 μg/kg FW in cultivar HS to 2919.09 ± 325.82 μg/kg FW in cultivar JNG (Tables S1 and S3).

### 3.3. Principal Component Analysis of Volatile Compounds

**Figure 3:**
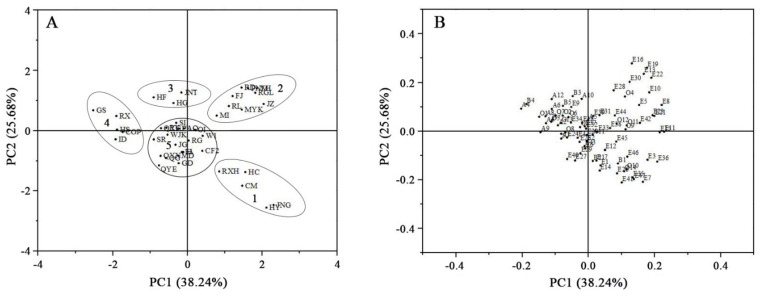
Principal component analysis (PCA) of 40 apple cultivars. (**A**) shows the PCA scores scatter plot. (**B**) shows a PCA loading plot. The codes in (**A**,**B**) correspond to codes in Table 1 and Table 2, respectively.

Principal component analysis (PCA), an unsupervised clustering method, is often used to provide a partial visualisation of data in a reduced-dimension plot [43,44]. PCA was used extract important information from the 78 volatile compounds detected in the 40 apple cultivars. As shown in Figure 3, the first two principal components accounted for 63.92% of the variation in the data, with PC1 and PC2 explaining 38.24% and 25.68% of the total variance, respectively. Scatter plots of the 40 apple cultivars are shown in Figure 3A, and the corresponding loadings establishing the relative importance of the variables are shown in Figure 3B. The 40 cultivars were divided into five groups based on the relationships between cultivars (scores) and their volatile compounds (loadings). The first group included five cultivars (RXH, JNG, CM, HY, HC), which contained high relative contents of butyl acetate (E7), hexyl acetate (E17), butyl caproate (E26), butyl heptanoate (E35), and estragole (O10). The second group contained eight cultivars (MI, FJ, RL, RD, MYK, JZ, RGL, PNM) characterised by high relative contents of 2-methylbutyl acetate (E8), amyl propionate (E13), 2-methylbutyl 2-methylbutyrate (E19), hexyl 2-methylbutyrate (E28), 2-methylbutyl hexanoate (E30), and 2-methylbutanoic acid (O4). The third group was composed of three cultivars (HF, HG, JNT), which contained high levels of propyl butyrate (E9), 2-methyl-1-butanol (B3), and 1-hexanol (B5). The fourth group included five cultivars (GS, HS, RX, COP, and ID) with low relative contents of esters and high relative contents of aldehydes such as 2-hexenal (A4), (E,E)-2,4-heptadienal (A9). The fifth group contained the other 19 cultivars, and showed no consistency in the composition of volatile compounds.

Among these cultivars, JNG in group 1 was characterised by high levels of butyl acetate (E7), hexyl acetate (E17), and butyl caproate (E26), in agreement with previous studies [25]. However, hexyl acetate (E17) was the major ester compound and was present in high levels in cultivar GD, which did not cluster into Group 1, possibly due to the influence of the content of other esters, such hexyl butyrate (E27) and hexyl hexanoate (E40). Cultivar FJ, one of the most widely cultivated apples in China, clustered into group 2 based on high relative content of 2-methylbutyl acetate (E8), amyl propionate (E13), 2-methylbutyl 2-methylbutyrate (E19), and hexyl 2-methylbutyrate (E28). 2-methylbutyl acetate is the main compound in the aroma profile of Fuji apples [45]. Granny Smith apples have low volatile emission compared with other apple varieties [46]. In this study, GS had low total content of volatile compounds, but the high relative content of 2-hexenal (A4) clustered it into group 4. As expected, group 5 contained the highest number of apple cultivars, and these cultivars had different types and contents of volatile compounds. These differences in the volatiles in cultivars contributed to diversity among apple varieties. According to PCA analysis results in this study, the most abundant esters in apple peels (hexyl butyrate, hexyl 2-methylbutyrate and hexyl hexanoate), together with hexanal, (E)-2-hexenal, 1-hexanol, estragole and α-farnesene could been proposed for apple cultivar classification in the future.

## 4. Conclusions

In this study, the identification, comparison and classification of volatile compounds in peels of 40 apple cultivars was carried out using HS-SPME combined with GC-MS. A total of 78 volatile compounds were detected in 40 apple cultivars. Eight volatile compounds were common in all the apple cultivars. Aroma profiles showed large differences among the cultivars. Cultivar Changfu No. 2 contained the highest number of volatile compounds, while Qinyue contained the least number of compounds. Honey Crisps had the highest volatile content, while Huashuo had the lowest volatile content. PCA clustered the 40 apple cultivars into five groups.

Overall, this study offered useful information for evaluating the profiles of volatile compounds in the peels of different apple cultivars and provided a reference for future breeding and improvement in apple flavour.

## Figures and Tables

**Table 1:** Apple cultivars used in this study and some basic fruit quality parameters.

No.	Cultivar	Code	SFW (g)	TSS (°Brix)	TA (%)
1	Royal Gala	RG	175 ± 15	12.7 ± 0.2	0.42 ± 0.03
2	Golden Delicious	GD	262 ± 22	13.5 ± 0.4	0.44 ± 0.04
3	Fuji	FJ	320 ± 25	13.2 ± 0.2	0.29 ± 0.02
4	Jonagold	JNG	280 ± 20	14.3 ± 0.3	0.37 ± 0.03
5	Indo	ID	350 ± 32	13.8 ± 0.2	0.13 ± 0.01
6	Orin	OI	255 ± 24	14.5 ± 0.1	0.30 ± 0.02
7	Hanfu	HF	285 ± 18	13.5 ± 0.2	0.36 ± 0.02
8	Jonathan	JNT	325 ± 23	14.1 ± 0.2	0.37 ± 0.03
9	Miyakiji	MYK	310 ± 26	14.6 ± 0.4	0.30 ± 0.03
10	Granny Smith	GS	285 ± 18	14.4 ± 0.3	0.37 ± 0.01
11	Ralls	RL	184 ± 12	14.0 ± 0.2	0.26 ± 0.02
12	Starkrimson	SR	275 ± 15	12.3 ± 0.1	0.28 ± 0.02
13	Huaguan	HG	178 ± 12	13.8 ± 0.3	0.27 ± 0.03
14	Huashuo	HS	266 ± 20	13.6 ± 0.2	0.38 ± 0.01
15	Huayu	HY	198 ± 12	13.1 ± 0.2	0.29 ± 0.03
16	Envy	EV	315 ± 24	14.6 ± 0.3	0.38 ± 0.02
17	Red General	RGL	268 ± 17	15.6 ± 0.3	0.28 ± 0.04
18	Starking	SI	290 ± 22	12.9 ± 0.2	0.32 ± 0.03
19	Jiguan	JG	217 ± 13	13.7 ± 0.1	0.26 ± 0.03
20	Cox Orange	COP	256 ± 20	13.2 ± 0.3	0.36 ± 0.02
21	Jazz	JZ	165 ± 10	12.2 ± 0.2	0.52 ± 0.05
22	Cameo	CM	334 ± 26	13.7 ± 0.4	0.39 ± 0.03
23	Honey Crips	HC	342 ± 28	14.0 ± 0.3	0.53 ± 0.05
24	Mollie’s Delicious	MD	280 ± 20	13.5 ± 0.2	0.29 ± 0.04
25	Modi	MI	195 ± 14	13.8 ± 0.3	0.42 ± 0.02
26	Qinguan	QG	332 ± 22	13.9 ± 0.1	0.16 ± 0.01
27	Qinyang	QYG	210 ± 15	12.1 ± 0.1	0.25 ± 0.01
28	Qinyue	QYE	182 ± 13	13.2 ± 0.2	0.29 ± 0.02
29	Qinyun	QYN	190 ± 14	13.3 ± 0.3	0.26 ± 0.01
30	World No.1	W1	510 ± 35	14.5 ± 0.3	0.26 ± 0.01
31	Weijieke	WJK	305 ± 25	12.8 ± 0.2	0.51 ± 0.04
32	Alps Otome	AO	50 ± 5	14.0 ± 0.2	0.27 ± 0.03
33	Red Delicious	RD	295 ± 15	13.5 ± 0.3	0.32 ± 0.02
34	Yuhuazaofu	YH	305 ± 16	13.4 ± 0.2	0.41 ± 0.03
35	Pink Lady	PL	183 ± 13	14.8 ± 0.3	0.52 ± 0.04
36	Changfu No.2	CF2	330 ± 26	15.4 ± 0.3	0.15 ± 0.01
37	Punama	PNM	246 ± 18	12.0 ± 0.2	0.28 ± 0.02
38	Ruixue	RX	296 ± 21	14.5 ± 0.2	0.30 ± 0.02
39	Ruiyang	RY	285 ± 20	13.5 ± 0.1	0.33 ± 0.03
40	Ruixianghong	RXH	165 ± 15	14.9 ± 0.2	0.24 ± 0.02

Datas are the mean value ± standard deviation of 9 samples (3 biological replicates × 3 technical replicates). SFW: Single fruit weight; TSS: total soluble solid content; TA: total acid content.

**Table 2:** Average contents of volatile compounds (*n* = 3, equivalent of 3-nonanone) and their distribution ranges (in parenthesis) in the peels of 40 apple cultivars.

Code ^a^	Compounds	CAS No ^b^	Odour Description ^c^	RT ^d^	RI ^e^/RI ^f^	Content (μg/kg FW)
Esters						
E1	Ethyl acetate	141-78-6	Pineapple, balsamic	8.74	894/893	3.01 (0–73.34)
E2	Ethyl propanoate	105-37-3	Banana, apple	10.20	964/964	0.24 (0–9.53)
E3	Propyl acetate	109-60-4	Celery	10.67	982/982	3.39 (0–52.92)
E4	Ethyl butyrate	105-54-4	Pineapple, fruity	12.33	1045/1048	8.52 (0–147.95)
E5	Propyl propionate	106-36-5	Fruity, sweet	12.57	1050/1045	2.43 (0–22.76)
E6	Ethyl 2-methylbutyrate	7452-79-1	Fruity, berry, fresh	12.78	1062/1063	4.85 (0–74.61)
E7	Butyl acetate	123-86-4	Fruity, ripe banana	13.40	1074/1075	145.31 (0–1064.54)
E8	2-Methylbutyl acetate	624-41-9	Fruity, banana	14.84	1126/1128	245.02 (0–1158.08)
E9	Propyl butyrate	105-66-8	Fruity	14.88	1135/1153	1.37 (0–26.05)
E10	Propyl 2-methylbutyrate	37064-20-3	Fruity, sweet	15.29	1150/1150	13.66 (0–94.23)
E11	Butyl propionate	590-01-2	Apple, fruity	15.41	1157/1158	51.57 (0–343.31)
E12	Amyl acetate	628-63-7	Pear, banana	16.37	1178/1185	20.15 (0–89.25)
E13	Amyl propionate	624-54-4	Fruity	16.84	1195/1208	11.01 (0–103.27
E14	Butyl butyrate	109-21-7	Fruity, apple, pear	17.69	1240/1240	103.51 (0–490.14)
E15	Butyl 2-methylbutyrate	15706-73-7	Fruity	18.08	1243/1241	204.79 (0–976.61)
E16	2-Methylbutyl butyrate	51115-64-1	Fruity	19.06	1270/1270	11.87 (0–69.21)
E17	Hexyl acetate	142-92-7	Sweet, flora, cherry	19.28	1274/1276	492.44 (10.61–1649.99)
E18	Pentyl valerate	2173-56-0	Fruity	19.45	1283/1284	3.59 (0–143.61)
E19	2-Methylbutyl 2-methylbutyrate	2445-78-5	Fruity	19.48	1286/1286	43.66 (0–268.76)
E20	Pentyl butyrate	540-18-1	Fruity	20.52	1321/1320	17.00 (0–81.93)
E21	Propyl hexanoate	626-77-7	Fruity, pineapple	20.58	1324/1324	10.08 (0–81.98)
E22	Amyl 2-methylbutyrate	68039-26-9	Fruity, apple	20.83	1330/1327	43.17 (0–180.78)
E23	Hexyl propanoate	2445-76-3	Fruity, sweet	21.14	1347/1344	180.82 (0–1404.90)
E24	Hexyl isobutyrate	2349-07-7	Fruity, sweet	21.18	1350/1353	14.55 (0–262.64)
E25	Heptyl acetate	112-06-1	Fruity, orange	22.09	1386/1386	0.30 (0–9.40)
E26	Butyl caproate	626-82-4	Fruity, acid, rancid	23.17	1410/1414	433.62 (7.70–1607.26)
E27	Hexyl butyrate	2639-63-6	Fruity, green, sweet	23.23	1423/1424	742.12 (3.62–2709.52)
E28	Hexyl 2-methylbutyrate	10032-15-2	Fruity, green	23.53	1438/1438	3085.20 (160.76–10087.55)
E29	Ethyl octanoate	106-32-1	Sweet, flora, pear	23.72	1445/1445	2.51 (0–44.50)
E30	2-Methylbutyl hexanoate	2601-13-0	Fruity	24.36	1467/1468	49.51 (0–291.37)
E31	trans-2-Hexenyl valerate	56922-74-8	Fruity	24.94	1478/1478	5.12 (0–57.99)
E32	Amyl caproate	540-07-8	Fruity	25.73	1508/1509	69.62 (0–364.73)
E33	Octyl hexanoate	4887-30-3	Fruity	25.75	1512/1512	12.29 (0–219.39)
E34	Hexyl valerate	1117-59-5	Fruity	25.77	1516/1516	8.48 (0–204.26)
E35	Butyl heptanoate	5454-28-4	Fruity	25.78	1518/1518	34.24 (0–363.05)
E36	Propyl octanoate	624-13-5	Fruity	25.93	1525/1525	7.68 (0–83.56)
E37	Heptyl valerate	5451-80-9	Fruity	26.10	1529/1530	4.72 (0–143.09)
E38	Heptyl 2-methylbutyrate	50862-12-9	Fruity	26.12	1530/1533	19.23 (0–111.43)
E39	3-methylbut-2-enyl hexanoate	76649-22-4	Fruity	27.53	1578/1575	0.37 (0–8.60)
E40	Hexyl hexanoate	6378-65-0	Fruity, wine	28.07	1593/1593	1444.76 (83.05–5688.01)
E41	Butyl caprylate	589-75-3	Slightly fruity	28.16	1603/1601	288.67 (0–2611.76)
E42	Hexyl tiglate	16930-96-4	Fruity	28.46	1631/1631	45.39 (0–233.71)
E43	Butyrolactone	96-48-0	Fruity	28.76	1638/1640	0.44 (0–17.42)
E44	2-Pentyl octanoate	55193-30-1	Fruity	29.11	1647/1645	12.12 (0–245.97)
E45	2-Methylbutyl octanoate	67121-39-5	Fruity	29.13	1648/1648	53.73 (0–320.66)
E46	Hexyl caprylate	1117-55-1	Fruity	31.82	1759/1760	114.96 (0–653.28)
E47	Butyl caprate	30673-36-0	Fruity	31.93	1765/1765	9.25 (0–163.04)
Aldehydes						
A1	Hexanal	66-25-1	Green, sweet	13.76	1090/1089	242.14 (29.29–1050.38)
A2	2-Methyl-4-pentenal	5187-71-3	Green	15.42	1156/1155	7.18 (0–110.33)
A3	(Z)-3-Hexenal	6789-80-6	Grass	15.60	1161/1158	8.11 (0–99.75)
A4	(E)-2-Hexenal	6728-26-3	Grass, herbaceous	17.93	1240/1220	2007.71 (569.95–4435.22)
A5	Octanal	124-13-0	Hone, green, fatty	19.85	1298/1298	2.13 (0–29.76)
A6	(Z)-2-Heptenal	57266-86-1	Grass	21.03	1339/1339	8.53 (0–53.46)
A7	Nonanal	124-19-6	Orange, grease	22.79	1401/1400	11.45 (0–96.12)
A8	(E)-2-Octenal	2548-87-0	Honey, green, fatty	23.89	1443/1441	2.61 (0–31.11)
A9	(E,E)-2,4-Heptadienal	4313-03-5	Cucumber	24.88	1497/1497	0.46 (0–9.32)
A10	(Z)-2-Nonenal	60784-31-8	Wet, fat, metallic	26.63	1531/1529	0.87 (0–23.49)
A11	Benzaldehyde	100-52-7	Sweet, fruity	26.66	1532/1532	5.41 (0–108.11)
A12	(E)-2-Decenal	3913-81-3	Sour, acidic	29.08	1655/1655	1.83 (0–38.62)
Alcohols						
B1	1-Propanol	71-23-8	Alcoholic	12.39	1048/1045	0.90 (0–35.99)
B2	1-Butanol	71-36-3	Sweet	15.35	1156/1158	25.14 (0–542.07)
B3	2-Methyl-1-butanol	137-32-6	Acidic, sharp, spicy	17.20	1210/1210	34.56 (0–149.4)
B4	2-Hexyn-1-ol	764-60-3	Green apple	17.41	1225/1223	22.98 (0–66.21)
B5	1-Hexanol	111-27-3	Unpleasant, green	21.40	1361/1361	82.66 (0–393.09)
Ketones						
C1	1-Penten-3-one	1629-58-9	Mushroom	12.07	1022/1020	1.45 (0–15.07)
C2	1-Octen-3-one	4312-99-6	Mushroom	20.22	1305/1305	3.74 (0–44.64)
C3	6-Methyl-5-hepten-2-one	110-93-0	Earthy, strawberry	21.26	1355/1348	3.38 (0–33.26)
Acids						
D1	2-Methylbutanoic acid	116-53-0	Fatty	29.37	1670/1670	38.34 (0–201.97)
Others						
O1	(E)-2-Pentenal	1576-87-0	Green	15.23	1142/1140	0.09 (0–3.78)
O2	Dodecane	112-40-3	Oily	16.79	1187/1187	6.96 (0–127.3)
O3	Tetradecane	629-59-4	Oily	22.49	1398/1398	30.38 (0–129.62)
O4	Copaene	3856-25-5	Woody, terpeny	25.56	1503/1505	2.88 (0–20.71)
O5	Hexadecane	544-76-3	Oily	27.59	1581/1581	12.00 (0–88.67)
O6	Estragole	140-67-0	Anise	29.66	1687/1687	293.26 (0–2012.56)
O7	α-Bergamotene	17699-05-7	Green	30.37	1694/1695	343.04 (0–1291.69)
O8	α-Farnesene	502-61-4	Green, oily, fatty	30.75	1725/1754	850.09 (7.76–2919.09)
O9	Thujopsene	470-40-6	Resinous	31.48	1747/1760	19.75 (0–97.63)
O10	Anethole	25679-28-1	Anise	32.40	1780/1780	47.93 (0–492.16)

^a^ Compound codes. ^b^ CAS number. ^c^ Odour description in the literature [25,26,27,28]. ^d^ Retention time (min). ^e^ Retention index in the HP-INNOWax column. ^f^ Retention index in the database (http://www.flavournet.org; http://webbook.Nist.gov/chemistry, accessed on 13 April 2021) and the literature [19,25,26,27,28]. FW: fresh weight

**Table 3:** Number of volatile compounds and total content of volatiles identified in the 40 apple cultivars.

No.	Cultivars	Number of Volatile Compounds	Total Content (μg/Kg FW)
1	Royal Gala	39	2919.26 ± 351.23
2	Golden Delicious	26	4436.74 ± 425.36
3	Fuji	42	3562.94 ± 310.02
4	Jonagold	38	23,047.24 ± 2826.62
5	Indo	36	5508.35 ± 401.23
6	Orin	30	16,863.94 ± 1806.24
7	Hanfu	38	10,988.51 ± 562.36
8	Jonathan	34	7436.91 ± 236.02
9	Miyakiji	38	12,817.30 ± 589.45
10	Granny Smith	27	3930.31 ± 328.94
11	Ralls	45	16,150.55 ± 2451.02
12	Starkrimson	29	3784.77 ± 327.05
13	Huaguan	34	12,184.76 ± 1087.69
14	Huashuo	21	2041.27 ± 120.36
15	Huayu	44	23,827.87 ± 3012.85
16	Envy	40	13,286.84 ± 1139.54
17	Red General	39	15,447.86 ± 1120.35
18	Starking	29	5411.64 ± 462.38
19	Jiguan	34	21,704.66 ± 1865.32
20	Cox Orange	24	2622.09 ± 150.74
21	Jazz	40	27,493.25 ± 3800.46
22	Cameo	36	20,118.58 ± 2010.38
23	Honey Crips	40	27,813.56 ± 2310.07
24	Mollie’s Delicious	29	5223.71 ± 362.38
25	Modi	44	12,564.23 ± 1835.44
26	Qinguan	32	26,132.20 ± 3450.20
27	Qinyang	33	5483.01 ± 280.74
28	Qinyue	20	4007.59 ± 263.58
29	Qinyun	27	10,963.94 ± 1021.56
30	World No.1	34	10765.78 ± 1806.75
31	Weijieke	25	5878.99 ± 350.28
32	Alps Otome	37	20,460.02 ± 1805.98
33	Red Delicious	37	14,524.14 ± 1205.32
34	Yuhuazaofu	40	8650.80 ± 680.21
35	Pink Lady	35	11,086.30 ± 1008.37
36	Changfu No.2	47	19,849.15 ± 2080.95
37	Punama	35	8480.92 ± 783.54
38	Ruixue	38	9274.25 ± 865.04
39	Ruiyang	25	4173.26 ± 280.86
40	Ruixianghong	43	27,015.38 ± 2540.92

**Table 4:** The total content (μg/kg) of each type of volatiles in peels of 40 apple cultivars.

Cultivars	Esters	Aldehydes	Alcohols	Others
RG	1691.11 ± 210.25	741.34 ± 80.43	94.28 ± 10.42	392.54 ± 32.51
GD	3240.04 ± 295.30	973.72 ± 85.62	44.02 ± 8.73	178.96 ± 20.98
FJ	2609.79 ± 252.76	662.21 ± 80.12	51.94 ± 4.38	239.01 ± 12.85
JNG	15,799.57 ± 1808.32	1900.45 ± 370.50	215.98 ± 20.84	5131.24 ± 486.22
ID	2542.49 ± 280.95	2467.84 ± 140.58	27.39 ± 3.85	470.63 ± 20.45
OI	12,073.00 ± 1500.65	3801.74 ± 364.02	94.56 ± 8.51	894.64 ± 107.84
HF	6242.30 ± 500.60	2487.06 ± 320.78	581.55 ± 42.89	1677.60 ± 137.21
JNT	3911.85 ± 410.20	2740.97 ± 200.36	215.97 ± 18.59	568.13 ± 46.25
MYK	8105.44 ± 742.39	1928.84 ± 200.45	143.13 ± 11.23	2639.89 ± 240.81
GS	504.68 ± 38.94	2650.55 ± 270.32	34.84 ± 5.20	740.25 ± 20.56
RL	11,716.86 ± 1520.36	2455.92 ± 325.60	150.24 ± 110.55	1827.52 ± 176.95
SR	1721.54 ± 160.98	1687.56 ± 200.85	18.63 ± 2.63	357.04 ± 40.28
HG	7797.49 ± 850.36	2961.00 ± 326.98	730.52 ± 50.46	695.76 ± 42.38
HS	474.23 ± 35.21	1475.38 ± 160.85	58.82 ± 5.96	32.84 ± 3.85
HY	14,491.84 ± 1628.32	2885.68 ± 203.56	941.15 ± 80.34	5509.20 ± 425.07
EV	10,691.41 ± 980.64	1317.54 ± 150.23	97.20 ± 10.55	1180.68 ± 140.36
RGL	10,167.00 ± 1230.52	2290.61 ± 180.56	184.35 ± 20.30	2805.91 ± 290.62
SI	1816.22 ± 178.21	2643.11 ± 250.36	26.48 ± 5.21	925.83 ± 86.33
JG	13,852.95 ± 1420.65	4905.73 ± 520.41	127.45±10.85	2818.53 ± 260.21
COP	608.05 ± 52.84	1815.17 ± 166.50	69.27 ± 6.21	129.60 ± 12.95
JZ	19,352.58 ± 1523.65	2509.39 ± 280.21	234.34 ± 19.85	5396.93 ± 500.42
CM	13,120.49 ± 1468.20	2642.90 ± 286.35	96.90 ± 8.55	4258.30 ± 480.74
HC	21,457.95 ± 2230.10	2609.31 ± 230.51	197.76 ± 15.42	3548.53 ± 384.19
MD	3270.42 ± 295.65	1359.24 ± 145.20	75.75 ± 8.52	518.30 ± 48.25
MI	8728.64 ± 865.32	2212.48 ± 284.50	103.19 ± 12.85	1519.92 ± 175.88
QG	19,396.06 ± 2010.57	4400.47 ± 385.12	121.38 ± 10.85	2214.29 ± 260.37
QYG	3036.79 ± 294.58	1842.62 ± 172.54	93.73 ± 10.25	509.86 ± 41.85
QYE	2684.64 ± 284.65	721.95 ± 85.24	214.52 ± 17.45	386.48 ± 33.06
QYN	7400.38 ± 851.54	1552.16 ± 160.22	177.79 ± 14.28	1833.61 ± 200.87
W1	6042.87 ± 576.25	2251.65 ± 280.35	48.54 ± 8.46	2422.72 ± 284.91
WJK	2465.30 ± 294.73	2525.87 ± 300.14	121.84 ± 10.85	765.97 ± 80.72
AO	14,748.22 ± 1624.35	2432.13 ± 281.45	397.55 ± 40.85	2882.11 ± 300.95
RD	8884.26 ± 960.35	3429.55 ± 302.85	65.59 ± 5.20	2144.75 ± 235.48
YH	5616.93 ± 596.21	2105.69 ± 248.52	122.03 ± 10.45	806.15 ± 67.58
PL	8144.07 ± 756.81	1684.40 ± 201.35	53.73 ± 5.21	1204.09 ± 82.13
CF2	15,358.65 ± 1742.23	2775.16 ± 208.95	175.76 ± 15.55	1539.57 ± 123.52
PNM	4374.62 ± 502.75	2657.88 ± 210.38	132.03 ± 15.20	1316.39 ± 150.70
RX	5215.59 ± 514.85	2815.66 ± 268.45	195.15 ± 20.96	1047.84 ± 82.09
RY	2454.10 ± 261.28	1434.19 ± 158.52	6.27 ± 1.02	278.70 ± 31.25
RXH	21,403.00 ± 2350.36	3182.62 ± 352.14	107.80 ± 80.56	2321.97 ± 213.50

## Data Availability

The datasets generated for this study are available on request to the corresponding author.

## Supplementary Materials

The following are available online at https://www.mdpi.com/article/10.3390/foods10051051/s1, Table S1: Number of apple cultivars for each identified volatile compound. Table S2. The content (μg/kg FW) of eight common volatile compounds in peels of 40 apple cultivars. Table S3: The contents (μg/kg FW) of identified volatiles in the peels of 40 apple cultivars. Table S4: Percentage (%) of each type of volatiles in apple cultivars.
